# Whole-body x-ray dark-field radiography of a human cadaver

**DOI:** 10.1186/s41747-020-00201-1

**Published:** 2021-01-26

**Authors:** Jana Andrejewski, Fabio De Marco, Konstantin Willer, Wolfgang Noichl, Alex Gustschin, Thomas Koehler, Pascal Meyer, Fabian Kriner, Florian Fischer, Christian Braun, Alexander A. Fingerle, Julia Herzen, Franz Pfeiffer, Daniela Pfeiffer

**Affiliations:** 1 Chair of Biomedical Physics, Department of Physics and Munich School of BioEngineering, Technical University of Munich, 85748 Garching, Germany; 2 Philips Research, 22335 Hamburg, Germany; 3 Institute of Microstructure Technology, Karlsruhe Institute of Technology, 76344 Eggenstein-Leopoldshafen, Germany; 4 Institut für Rechtsmedizin, Ludwig-Maximilians-Universität München, 80336 Munich, Germany; 5 Department of Diagnostic and Interventional Radiology, Technical University of Munich, 81675 Munich, Germany

**Keywords:** Dark-field imaging, Human body, Radiography, Whole-body imaging, X-rays

## Abstract

**Background:**

Grating-based x-ray dark-field and phase-contrast imaging allow extracting information about refraction and small-angle scatter, beyond conventional attenuation. A step towards clinical translation has recently been achieved, allowing further investigation on humans.

**Methods:**

After the ethics committee approval, we scanned the full body of a human cadaver in anterior-posterior orientation. Six measurements were stitched together to form the whole-body image. All radiographs were taken at a three-grating large-object x-ray dark-field scanner, each lasting about 40 s. Signal intensities of different anatomical regions were assessed. The magnitude of visibility reduction caused by beam hardening instead of small-angle scatter was analysed using different phantom materials. Maximal effective dose was 0.3 mSv for the abdomen.

**Results:**

Combined attenuation and dark-field radiography are technically possible throughout a whole human body. High signal levels were found in several bony structures, foreign materials, and the lung. Signal levels were 0.25 ± 0.13 (mean ± standard deviation) for the lungs, 0.08 ± 0.06 for the bones, 0.023 ± 0.019 for soft tissue, and 0.30 ± 0.02 for an antibiotic bead chain. We found that phantom materials, which do not produce small-angle scatter, can generate a strong visibility reduction signal.

**Conclusion:**

We acquired a whole-body x-ray dark-field radiograph of a human body in few minutes with an effective dose in a clinical acceptable range. Our findings suggest that the observed visibility reduction in the bone and metal is dominated by beam hardening and that the true dark-field signal in the lung is therefore much higher than that of the bone.

## Key points

- We presented the first whole-body x-ray dark-field images of a human body.
- In the used setup, dark-field signal of the bones originates mainly from beam hardening.
- Beam hardening correction methods are important for medical applications of x-ray dark-field radiography.

## Background

Besides the x-ray attenuation, which is measured by conventional x-ray imaging, wave-optical effects such as refraction and small-angle scatter of x-rays occur on interaction with matter.

X-ray phase-contrast imaging utilises a Talbot-Lau interferometer to measure these effects. By placing three gratings in the beam path of a conventional source, intensity modulations are created from which the phase and small-angle scatter information can be obtained. Subsequently, attenuation, differential phase, and dark-field images of a sample placed in the beam path can be calculated form these recorded modulations [1–10]. The dark-field image provides a measure for the magnitude of small-angle x-ray scattering induced by a sample. The correlation between dark-field signal and microscopic sample parameters has been examined in detail [11–13].

A conventional source has a broad x-ray energy spectrum, and thus, beam hardening occurs when measuring thicker samples. This spectral change also affects the dark-field measurement, as the dark-field signal is highly dependent on the x-ray energy *E* [11, 13, 14].

Applications for this new imaging modality have been found both in the context of basic research, non-destructive testing, and medical diagnostics [15–29]. Concerning the latter, the first *in vivo* dark-field radiograph of a mouse revealed a high dark-field signal originating from the lungs [30], which motivated a large number of small animal studies as many pulmonary diseases are related to structural impairments. These determined a significant potential of dark-field radiography and computed tomography (CT) for the detection of structural pulmonary diseases [31–42]. A further important step towards clinics was the development of x-ray dark-field systems allowing imaging studies of the lung of larger animals and human bodies [17, 43–46]. Beyond the lung, the internal structure of bones was also recognised to be a source of dark-field signal [47, 48]. One study [43] presented full body x-ray dark-field and transmission images of a euthanised pig. The authors found strong dark-field signal in the lung as well as in some skeleton structures.

We present transmission and dark-field images covering the majority of a human body. We compare the signal strengths of both types of images for different body regions and discuss the detectability of anomalies on both image modalities. Furthermore, the effect of beam hardening on dark-field images is discussed by comparing the dark-field signal of different scattering and absorbing materials.

## Methods

### Imaging setup

The three-grating setup has been previously described [44–46]. The source grating (*G*_*0*_) had an area of 5.0 × 2.5 cm^2^, a period of 68.72 μm, and a duty cycle of 0.7. The other two gratings (*G*_*1*_: period 8.73 μm, duty cycle 0.5, *G*_*2*_: period 10 μm, duty cycle 0.5) were created from eight tiles, each with a size of 5.0 × 2.5 cm^2^, to achieve a total size of 40 × 2.5 cm^2^ per grating, as already described by Schröter et al. [49]. The *G*_*0*_*–G*_*1*_ and the *G*_*1*_*–G*_*2*_ distances were 1.60 and 0.25 m, respectively. All gratings were gold-filled attenuation gratings (gold filling height of 150–200 μm) provided by Karlsruhe Institute of Technology (Karlsruhe, Germany) and Microworks GmbH (Karlsruhe, Germany). For this highly asymmetric configuration, the immediate shadow of *G*_*1*_ instead of the Talbot effect was exploited to generate an intensity pattern at the position of *G*_*2*_. A similar approach was already described [50]. All gratings are mounted on a common frame pivoted about an axis through the source’s focal spot. An effective field of view of 32 × 35 cm^2^ was achieved (in the object plane, 10 cm above the sample table) by scanning the grating frame across the detector. Source, detector and sample remain stationary during the acquisition. Acquisition time was 40 s per scan. The x-ray source (Philips MRC 200 0310 ROT GS, Philips Medical Systems, Hamburg, Germany) was operated at 70 kVp and 700 mA for the whole-body measurement.

### Data acquisition and processing

For the acquisition of the individual images, a moiré fringe-scanning method was used [17, 51]. By introducing a slight mismatch of the distance between modulation grating and analyser grating, a low-frequency fringe pattern is generated in the detector plane, representing the relative lateral positions of modulation and analyser grating structures. This pattern is scanned over each sample point via movement of the grating frame, generating a series of images. In this way, a moiré pattern is sampled for each detector pixel, and a method similar to the more common, so-called phase-stepping acquisition procedure [8, 17] can be used to retrieve mean intensity *A*_*s*_, interferometric visibility *V*_*s*_ (*i.e*., the relative contrast of the fringe pattern), and lateral phase shift *φ*_*s*_ of the pattern. Repeating the procedure without a sample yields the reference values *A*_*r*_, *V*_*r*_, and *φ*_*r*_, allowing the calculation of attenuation *α* = *− ln (A*_*s*_*/A*_*r*_*)* and visibility reduction *ν* = *− ln (V*_*s*_*/V*_*r*_*)* of the sample.

Since a visibility reduction due to beam hardening occurs in areas with high attenuation, a correction method, adapted from Pelzer et al. [52], was used to rectify the dark-field signal: transmission *T*_*k*_ and visibility reduction *ν*_*k*_ signals were measured for several polyoxymethylene (POM) plastic heights *h*_*k*_. A reference curve *ν*_*BH*_*(T)*, which indicates visibility reduction as a function of transmission, was calculated from these points. The corrected dark-field signal *D* of a sample is then given by *D* = *ν − ν*_*BH*_. POM was chosen as a reference material for soft tissue and fat in the visibility reduction calibration as it has similar spectral attenuation properties as soft tissue and fat.

The transmission and dark-field images of the human body were combined from six individual scans, leading to a total scanned area of 177 × 30 cm^2^ in the patient plane. The body was placed on a motorised sample table. The upper body was imaged in three scans, and the scan range was selected by adjusting the table position before each scan. Due to the table limited movement range, the body was then rotated by 180° to allow imaging the lower body. Three additional scans were acquired in this orientation.

### Human cadaver

The experiment was approved by the institutional review board (Ethikkommission der Ludwig-Maximilians-Universität München, Pettenkoferstraße 8a, 80336 Munich, Germany) and was conducted according to the Declaration of Helsinki. The full-body radiographs were taken from a 62-year-old female body (height 162 cm, weight 49 kg). Images were taken 4 days postmortem, and the lung was ventilated by an anaesthesia machine (Fabius® Tiro, Drägerwerk AG & Co. KGaA, Lübeck, Germany) with a constant pressure of 18 mbar.

### Dose estimation

The dose area product was estimated from the incident air kerma, measured with a PTW NOMEX dosimeter (PTW, Freiburg, Germany) and the area of the organ. With this, the effective dose (ED) for different organs can be calculated using conversion factors given by Wall et al. [53].

### Beam hardening measurements

In the experiment determining the effect of beam hardening on the x-ray dark-field signal, different heights of POM, aluminium, and neoprene were measured in a phantom experiment as well as an *ex situ* pig lung obtained from a local butcher. For the values of aluminium, POM and neoprene, the transmission and dark-field signal was calculated from the mean signal across a region of interest of 22 × 47 mm^2^ for each material height. The pig lung was ventilated with a pressure of 25 mbar. Each point in the provided scatter plot corresponds to a value pair (T, ν) of one pixel in the two (registered) images (see Fig. 4).

### Data presentation

Data are given as simple point values or estimation or as mean ± standard deviation.

## Results

**Fig. 1:**
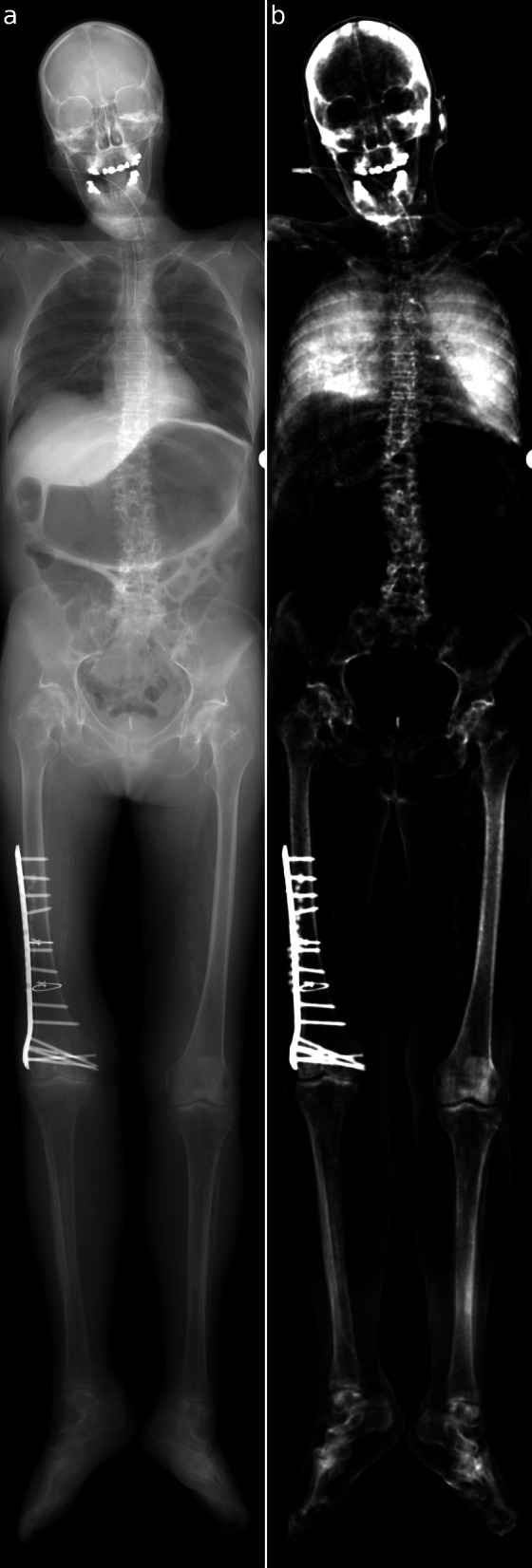
Attenuation (window level 2.5, window width 5) (**a**) and x-ray dark-field (window level 0.175, window width 0.35) (**b**) radiographs of a human cadaver excluding the arms, recumbent, anteroposterior acquisition with the right hand side of the body on the left side. The images are created from six individual scans. A correction for visibility reduction due to beam hardening was applied

**Fig. 2:**
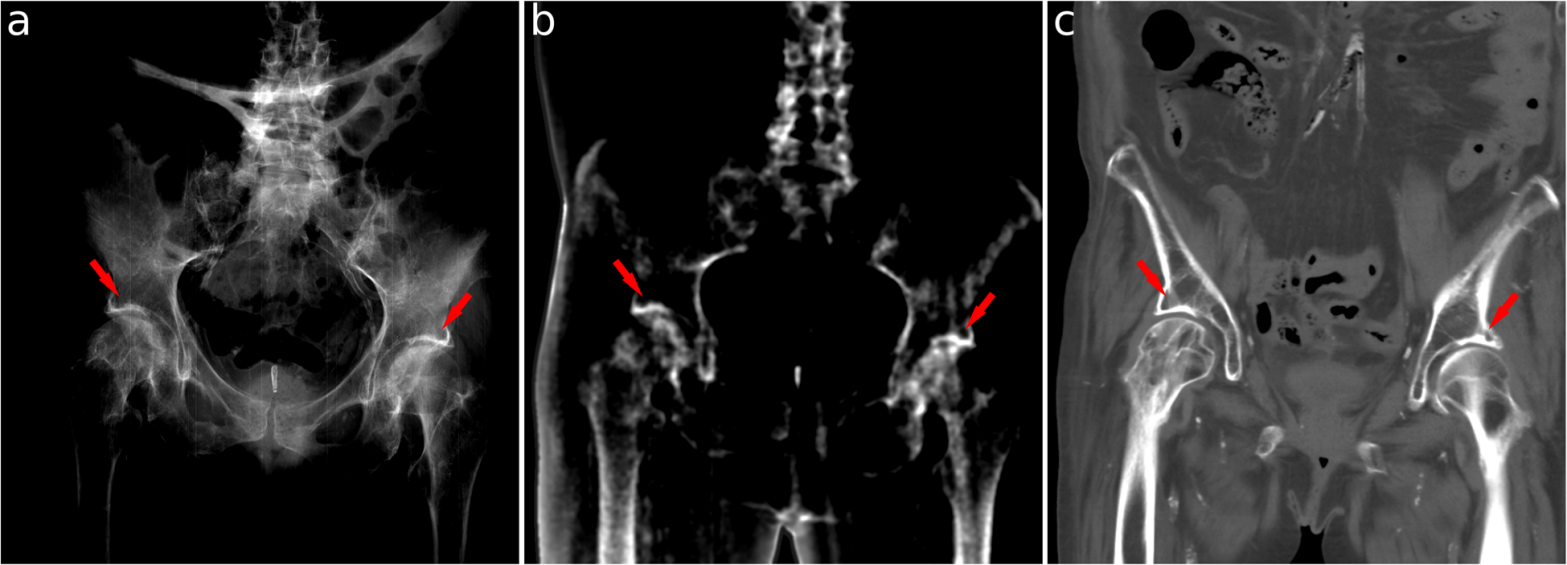
Conventional x-ray (window level 4.05, window width 1.1) (**a**) and dark-field radiograph (window level 0.8, window width 2.0) (**b**), and coronal computed tomography reconstruction (**c**) of the pelvic region. The red arrows mark an area with subchondral sclerosis as a cause of bilateral osteoarthritis of the hips. These areas exhibit an increased signal at both modalities. Whereas the increased attenuation signal is due to the associated thickening of the bone structure, the increased dark-field signal can be attributed due to spectral hardening effects as well as increased small-angle scatter

**Fig. 3:**
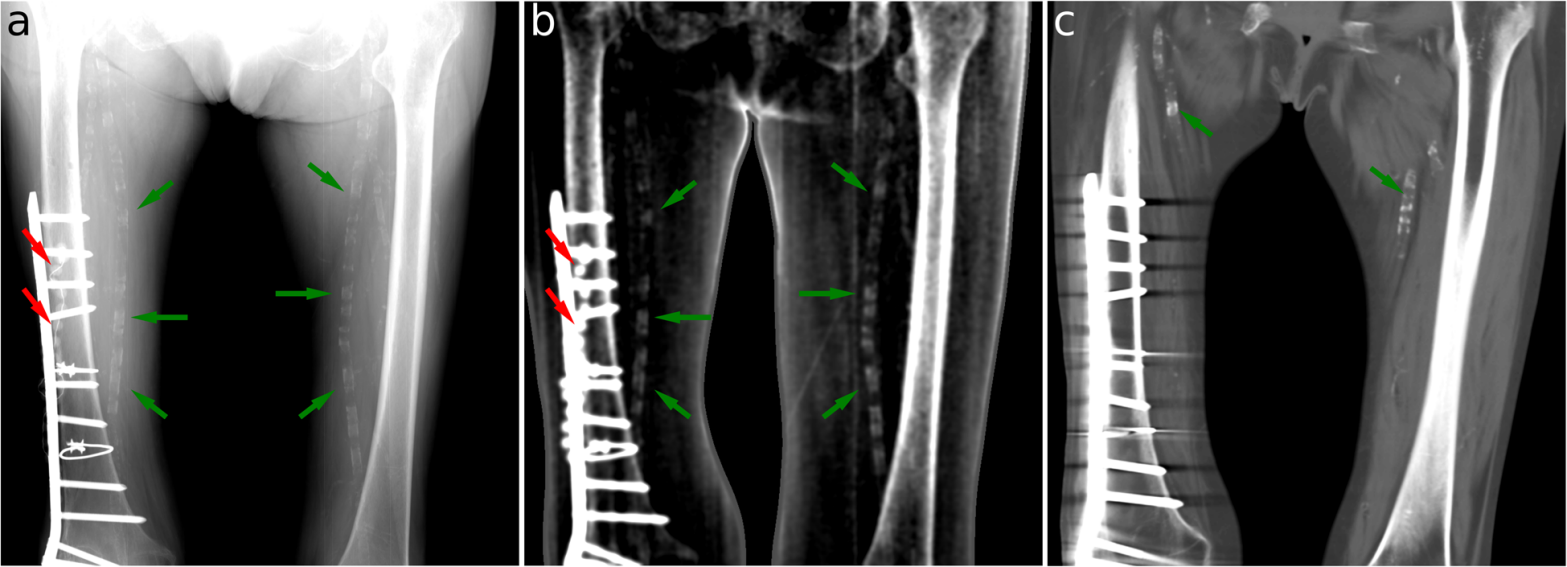
X-ray attenuation (window level 2.45, window width 1.9) (**a**), dark-field (window level 0.4, window width 2.0) (**b**) radiographs, and a computed tomography reconstruction (**c**) of the upper legs. The metal screws and plate on the right femur, a fixation of an old fracture, are clearly visible in all three modalities. Near the screws, an antibiotic bead chain is also visible in the attenuation and dark-field images (red arrows). Furthermore, calcifications are visible within the superficial femoral arteries in both thighs at all three modalities (green arrows)

Full-body transmission and dark-field images of a human body are shown in Fig. 1. These images were combined from six individual scans. Figures 2 and 3 show transmission and dark-field radiographs of the hip and the thigh regions with a narrower windowing in order to highlight features in these regions.

For different organs, a rough estimation of ED is given in Table 1. For comparison, typical ED reference values as reported by Wall et al. [53] are presented for these organs. The ED values for the chest, knee, and feet were a lot higher than the typical ED values, whereas the ED values for the head, abdomen, and pelvis were in the range of the typical ED values.

**Table 1:** Rough estimation of effective dose in the study and literature values for different organs

Organ	ED (mSv)	Typical ED (mSv)^a^	ED/typical ED
Head AP	0.034	0.033	1.03
Chest PA	0.268	0.014	19.14
Abdomen AP	0.300	0.430	0.70
Pelvis AP	0.220	0.280	0.79
Femur AP	0.051	0.011	4.64
Knee AP	0.004	0.0001	40.00
Feet oblique	0.0024	0.0001	24.00

*AP* Anteroposterior, *PA* Posteroanterior, *ED* Effective dose

^a^Wall et al. [53]

The inflated lungs exhibit high transparency in the attenuation image. In the dark-field image, both lungs reveal a high signal (0.25 ± 0.13). The highest signal intensity was seen in the lower lobe of the right lung with 0.34. Conversely, low transmission signal and no dark-field signal are observed in the abdominal region. Bony structures are visible in the attenuation as well as in the dark-field images. The mean dark-field signal of the bones was 0.08 ± 0.06, with a maximum of 0.23 in the cortical bone of the upper left leg. In particular, an increased signal in both modalities occurs in the hip joints, where anatomical changes due to osteoarthritis are present (see Fig. 2). Furthermore, the right leg presents the result of osteosynthesis following a fracture of the right femur. The fracture itself is not visible anymore, which correlates nicely with the CT image, where the complete healing of the fracture can be confirmed. In contrast, the metal screws and plate are clearly visible. In addition, an antibiotic bead chain, which is still *in situ*, is clearly visible in the dark-field image (0.30 ± 0.02). With respect to vascular structures, the calcification of the femoral arteries can be clearly depicted in both the upper legs in all three modalities (see Fig. 3). Finally, in soft tissue, barely any dark-field signal can be observed (0.023 ± 0.019).

**Fig. 4:**
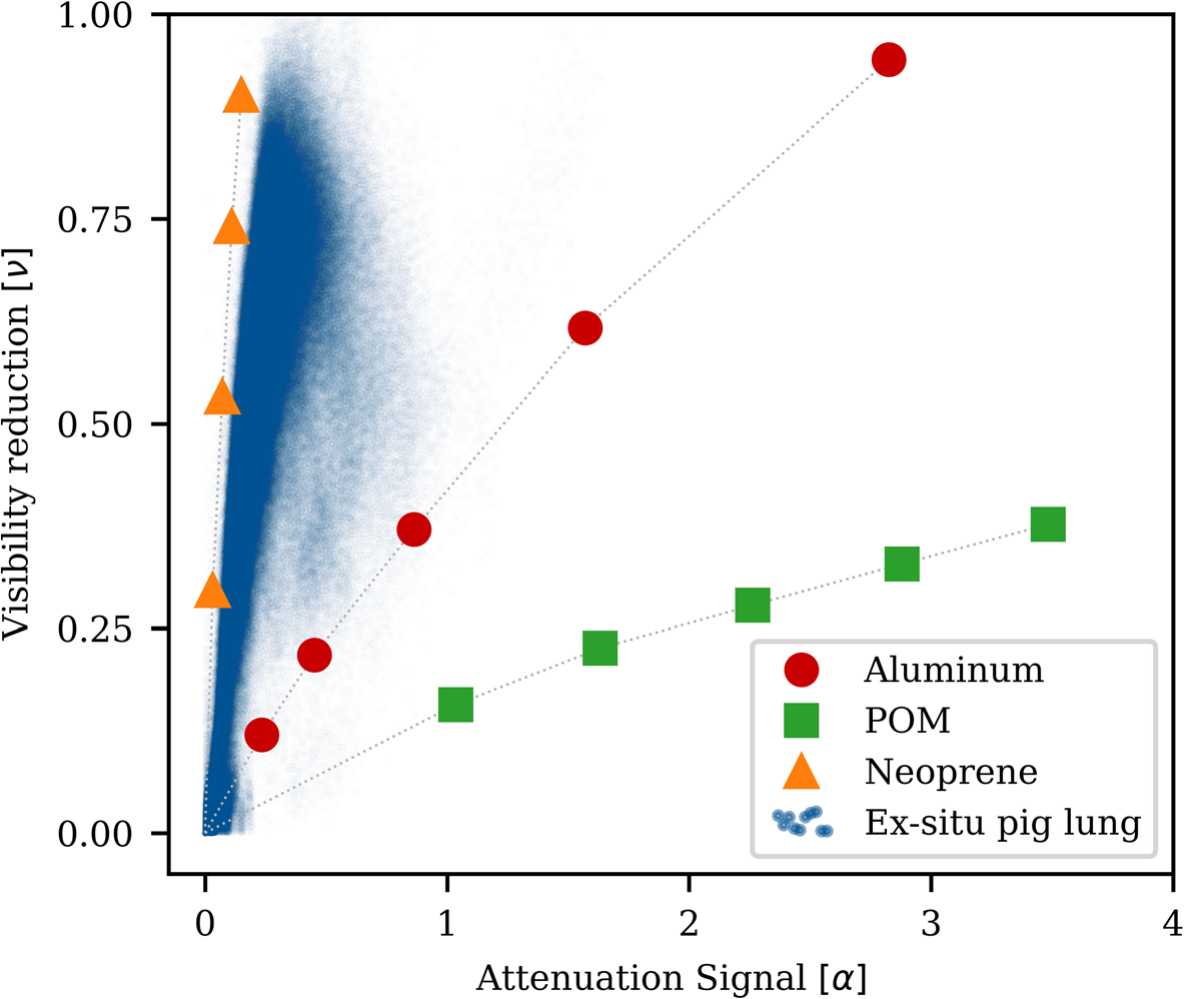
Effect of beam hardening and small-angle scattering on the x-ray dark-field signal. Measured with 1, 2, 4, 8, and 16 mm of aluminium; 3, 5, 7, 9, and 11 cm of polyoxymethylene (POM); 1, 2, 3, and 4 cm of neoprene and an *ex situ* pig lung. Although no scattering object was in the beam path during the measurement of POM and aluminium, an apparent dark-field signal was generated by beam hardening. Aluminium exhibits similar spectral attenuation properties as the bone, whereas POM more closely approximates soft and adipose tissue. The large discrepancy between the two curves explains the strong apparent dark-field signal due to the bones in Figs. 1, 2, and 3, despite application of a POM-based beam hardening correction

Figure 4 shows a plot of uncorrected visibility reduction *ν* = *−ln (V*_*s*_*/V*_*r*_*)* *versus* the corresponding attenuation signal *α* = *− ln (A*_*s*_*/A*_*r*_*)* of each measured phantom material (*i.e*., aluminium, POM, neoprene and an *ex situ* pig lung). It is apparent that the visibility reduction signal of the lung and neoprene increases more steeply as a function of the transmission signal than that of aluminium. Among all measured materials, the visibility reduction signal of POM increases the least with *α*. For POM and aluminium, the transmission signal increases as well as the visibility reduction, even though these objects do not produce small-angle scatter. The increase of visibility reduction is stronger for aluminium than for POM.

## Discussion

In past small animal studies, x-ray dark-field imaging showed a potential benefit in diagnosing lung diseases [31–42, 54]. Subsequent large animal and human cadaver measurements showed the possibility to translate x-ray dark-field radiography to the human scale [43–46], and a first study on dark-field chest radiographs of human bodies showed that the dark-field signal could be reliable quantified [55]. However, further applications of x-ray dark-field imaging has not been extensively investigated on large animal models or human cadavers yet.

In this study, we presented the first x-ray dark-field image of a complete human body and compared findings in the transmission, dark-field and CT images. A high dark-field signal was found in the lung. Furthermore, the bone, calcification in the femoral arteries, implants and foreign bodies also produce a dark-field signal. Phantom measurements showed that a strong visibility reduction signal can be produced by objects which do not generate small-angle scatter.

In this study, the ED values resulted to be higher than typical ED in some organs. The typical ED are given for radiographs where only the organ in question is imaged. As a consequence, image parameters like tube current were chosen to be optimal for imaging this organ. In our study, the imaging parameters were the same for all organs. Thus, the tube current had to be chosen in such a way that enough photons reach the detector behind the more absorbing body parts like head, pelvis, and abdomen. For those regions, the ED in this study resulted to be similar to the typical ED. Higher ED in less absorbing organs is a consequence of this experimental setup.

Our results concerning the thorax region are similar to the results reported in other studies [43, 44, 46]. The considerable dark-field signal in the lung originates from small-angle scattering on the numerous air-tissue interfaces in the lung parenchyma, whereas the high transmission is due to the weak attenuation of the mainly air-filled lungs. In this study, the dark-field signal of the lung was lower compared to the dark-field signal of the pig lung reported by Hauke et al. [43]. The authors euthanised a pig a few minutes before image acquisition, whereas in this study, the images were taken 4 days postmortem. Therefore, the lung of the human body was more strongly collapsed with partially fluid-filled alveoli compared to the pig lung and not as many air-tissue interfaces were present anymore in the human lung.

In contrast to the lungs, barely any air-tissue interfaces exist in the stomach and, as a consequence, no dark-field signal originates in the stomach. The low attenuation signal is a result of the stomach being extensively filled with air due to a previous incorrect positioning of the endotracheal tube within the oesophagus.

Dark-field signal is generated by the individual beads of the antibiotic bead chain, as these are made from a spongious material which acts as a substrate for the antibiotic agent. Spongious materials contain large numbers of interfaces with air, leading to an increase in scattering and therefore to an increased dark-field signal.

Calcification, *i.e.,* accumulation of calcium salt, in particular of arterial walls (as it can be seen in atherosclerosis), is a common finding, especially in patients with cardiovascular risk factors. Atherosclerosis can lead to stenosis of the affected arteries, resulting in hypoperfusion of the anatomical region which is supplied by the respective artery. Depending on the affected arteries, patients can suffer from coronary or peripheral artery disease, as it was for the patient in our study. Previous studies showed that calcification in the breast is visible in the dark-field image, potentially improving breast cancer detection [56–58]. We were also able to see calcification of the arteries in the dark-field image. However, whether the calcification is better and earlier visible on dark-field than common transmission images and if dark-field imaging has the potential to improve the diagnostic value has to be determined in further studies.

Bony structures are visible in both transmission and dark-field images, especially the areas of increased calcium content: subchondral sclerosis as a cause of bilateral osteoarthritis of the hips was highly visible in the dark-field image. Similarly, subchondral sclerosis due to osteoarthritis of both knees also resulted in a high signal on the dark-field images.

As visible in Fig. 4, even non-scattering materials can cause a reduction in visibility which is due to beam hardening. The interferometric visibility of an x-ray dark-field imaging setup is not only dependent on the setup arrangement (*i.e.,* grating periods and inter-grating distances), but also on the spectrum of the x-ray beam. In our setup, visibility was high for photon energies up to 40 keV and decreases continuously for higher energies. When measuring with energy integrating detectors, the average visibility results from the detector signal of the energy dependent visibility weighted with the detected signal from each photon energy [59]. Since the low-energy x-rays, which contribute the most to the measured visibility, are preferentially attenuated by most materials, the measured, average visibility decreases. As a result, non-scattering, strongly attenuating materials can induce a decrease of visibility, which is indistinguishable from a true dark-field signal due to small-angle x-ray scatter. For soft and adipose tissues, this effect could largely be eliminated by a correction, which is based on visibility reduction measurements of POM. As POM has spectral attenuation properties similar to soft tissue, which differs significantly from those of bone and metal, the correction however fails to accurately model their contribution to beam hardening, and a residual visibility reduction signal remains.

It has been shown that the bones also generate a dark-field signal [47, 48, 60]. The dark-field signal of the bones is lower as the signal of the lungs. Our setup showed a lower sensitivity than the ones used for bone measurements. Therefore, in our opinion, the dark-field signal of the bones in the here presented measurements mainly result from residual visibility reduction signal.

Our study has some limitations. First, only one human body was imaged. Furthermore, the complete image was stitched together from six individual scans and the body had to be repositioned during the acquisition procedure. This experimental setup is not practical as an imaging approach in clinical routine. This problem could be overcome by changing the acquisition of the dark-field images: instead of moving the interferometer, the sample could be moved as proposed by Seifert et al. [61]. Thus, the patient can be imaged in one scan similar to a full body CT scan. Lastly, the beam hardening correction was performed with respect to the soft tissue component. Removal of beam-hardening-related artefact depends on the knowledge of the material composition of the body part in question. Calibration data and algorithms used for correction must therefore be tailored to the measured body part. In particular, superposition of materials with very different spectral properties (*e.g.,* bone and soft tissue) presents a special challenge for beam-hardening-related visibility correction in dark-field radiography, since the individual material contributions to the total attenuation cross-section along the x-ray path are unknown and vary over the field of view. A useful approach in such a case may be to use dual-energy acquisition, which may allow calculating spatial maps of such cross-section fractions and thus enabling correction of beam-hardening-related dark-field artefact for two different materials.

In conclusion, we gave an overview over the dark-field signal strength of different organs of the human body presenting images and data from a female cadaver. However, before dark-field imaging can be included into the clinical routine, further studies will have to be conducted. Besides lung imaging, bone imaging and the possibility of diagnosing calcification might be of special interest.

## Data Availability

The datasets used and/or analysed during the current study are available from the corresponding author on reasonable request.

## Abbreviations

CT: Computed tomography
ED: Effective dose
POM: Polyoxymethylene
